# Developing Interim Outcome Measures for Multi-Arm Multistage Clinical Trials in Multiple Sclerosis

**DOI:** 10.1212/WNL.0000000000218426

**Published:** 2026-09-24

**Authors:** Sean Apap Mangion, Charles Wade, Daniel Coles, Rachel Burton, Jennifer M. Nicholas, Douglas Lorne Arnold, Arman Eshaghi, Jeremy Chataway

**Affiliations:** 1 Queen Square Multiple Sclerosis Centre, Department of Neuroinflammation, UCL Queen Square Institute of Neurology, Faculty of Brain Sciences, University College London, United Kingdom;; 2 University College London Medical School, Faculty of Medical Sciences, University College, United Kingdom;; 3 Medical Research Council Clinical Trials Unit, University College London, United Kingdom;; 4 London School of Hygiene & Tropical Medicine, Centre of Global Change and Health, Medical Statistics, United Kingdom;; 5 Brain Imaging Centre, Montreal Neurological Institute and Hospital, McGill University, Quebec, Canada;; 6 UCL Hawkes Institute, University College London, United Kingdom; and; 7 National Institute for Health and Care Research, University College London Hospitals Biomedical Research Centre, United Kingdom.

## Abstract

**Background and Objectives:**

Multi-arm multistage (MAMS) clinical trials use interim outcome stage analyses to test multiple candidate treatments, determining which should proceed to the next trial stage. The reduced time, cost, and participant numbers, compared with current separated phases 2 and 3 clinical trials, could expedite the development of treatments in neurodegenerative diseases, such as progressive multiple sclerosis (PMS). MAMS trial interim outcomes must reflect the final stage outcome and be able to detect treatment effects with high power at interim stages. Patient-reported outcomes (PROMs) are cost-effective and highly accessible, making them promising MAMS trial interim outcome measures. We determined whether the MS Quality of Life 54 Physical Health Composite, MS Impact Scale 29 version 2 physical health subscore (MSIS-29v2 PH), MS Walking Scale 12 version 2 (MSWS-12v2), or their composite could act as interim outcome measures in MAMS PMS trials.

**Methods:**

Using a pooled analysis of 11,052 longitudinal visits from 3 PMS trials, we assessed the relationship of PROM change at early time points with 6-month confirmed disability progression (CDP) in the Expanded Disability Status Scale (EDSS) up to 36 months through Cox proportional hazards. To minimize PROM ceiling effects, we generated an MSIS-29v2 PH and MSWS-12v2 composite, prioritizing greatest change. We calculated the required sample size when these PROMs were used as interim measures.

**Results:**

We analyzed 1,927 of the 2,046 available participants (mean age 54 [SD 7.2] years and 66% female). EDSS 6-month CDP risk was significantly associated with each 1-standard deviation 12-month score increase for the MSIS-29v2 PH (hazard ratio [HR] 1.20 [1.08–1.33]), MSWS-12v2 (HR 1.26 [1.13–1.40]), and composite (HR 1.29 [1.16–1.44]; all *p* < 0.0001). In simulations, using the composite, n = 125/arm can detect a 50% treatment effect on mean 12-month change with power ≥90% and 200/arm with a power ≥95%.

**Discussion:**

Changes in the MSIS-29v2 PH, MSWS-12v2, and composite scores associated with ≥6-month EDSS CDP up to 36 months. The composite has sufficient statistical power to detect treatment effects at 12 months. These PROMs are well placed to play a role in interim analysis in MAMS trials in PMS.

## Introduction

The development path for neurodegenerative diseases is slow and expensive, with a high rate of failure.^1-3^ It is a major unmet need.^4-6^ Platform multi-arm multistage (MAMS) trials are 1 way to expedite this process by testing multiple drug arms with shared controls and assessing treatment effects at interim analyses, thereby reducing time, cost, and participant numbers.^7^ They have been successful in infectious diseases and oncology.^8,9^

The focus of interim stage analyses is on identifying which treatment arms are unlikely to show a treatment benefit on the final (phase 3) stage outcome.^10^ The selected interim outcome measure must therefore relate to it and be able to demonstrate a treatment effect in a smaller sample size and at the chosen interim time point with sufficient statistical power.^11^

In progressive multiple sclerosis (PMS), the MAMS-design OCTOPUS trial (ISRCTN14048364) uses 2 outcome analysis stages, stage 1 interim and stage 2 final, and is targeting putative neuroprotective compounds, currently either metformin or R/S-alpha lipoic acid against placebo.^12^ Previous work^11^ demonstrated that MRI whole-brain atrophy (WBA) can reasonably be used as the stage 1 interim outcome both from a biological and statistical perspective. Biologically, it reflects the pathologic processes of disability progression and has a treatment effect size which correlates with trial relevant clinical disability measures. Statistically, the allowance of a higher false-positive treatment effect at this interim stage provides a high degree of certainty (≥95%) that any ineffective treatment arms can be reliably dropped. However, the use of MRI WBA comes with limitations: high associated costs, signal loss from scanner changes, and with potential tolerability issues for participants.^4,13-15^ Therefore, there is a need to develop other interim outcomes, either to replace or augment WBA measures.

There is a growing interest in patient-reported outcome measures (PROMs), by the Food and Drug Administration^16^ and European Medicines Agency.^17^ PROMs are tools that capture a patient's perception of their health-related quality of life (QoL)^18^ and are used in policy making decisions.^19,20^ They are practical, economical, amenable to remote collection, and have evident associations with clinical outcomes.^21-24^ Examples in MS are the MS-QoL-54,^25^ MS Impact Scale 29 version 2 (MSIS-29v2),^26,27^ and MS Walking Scale version 2 (MSWS-12v2).^28,29^ Although these instruments share some overlap in assessing physical function and activities of daily living, they differ in scope and specificity. The MS-QoL-54 was adapted from a generic PROM and its physical health composite (PHC) subscore captures broad health-related quality of life, incorporating items spanning upper and lower limb function across different contexts and degrees of physical intensity (such as vigorous vs moderate activities, climbing stairs, walking distances, and lifting/carrying objects), as well as additional questions around social, occupational, and sexual function.^25^ The MSIS-29v2 physical health (PH) subscore^26,27^ was developed to assess MS-specific physical impact, with items that broadly enquire about difficulties in function of both upper and lower limbs, social or physical activities, or the ease with which physical function is achieved. The MSWS-12v2 is also MS specific but focuses explicitly on mobility,^28,29^ with appreciably more granular items around walking, standing, climbing stairs, and the use of mobility aids indoors and outside. The MS-QoL-54 PHC, because of its broader scope and greater number of constituent items, shares the greatest overlap with both the MSIS-29v2 PH and MSWS-12v2. A post hoc analysis of a phase 3 trial of siponimod in secondary PMS (SPMS) highlighted their potential for early treatment effect detection, having demonstrated a 1 year mean change difference of 70% in the MSIS-29v2 PH subscore, and 55% in the MSWS-12v2, compared with a 21% risk reduction in clinical disability progression up to 3 years.^30,31^ This combination of regulatory support, patient centeredness, economic advantages, and evidence of early treatment effects makes them especially appealing as interim measures in MAMS trials.

Therefore, we aimed to assess whether PROMs could act as interim outcome measures in a MAMS trial in PMS by pooling data from 2 phase 2 and a phase 3 clinical trial of over 2,000 participants. Our first objective was to determine whether earlier changes in PROMs could reflect longer-term clinical disability. Our secondary objective was to establish the sample sizes required to reliably detect putative treatment effects in MAMS trials.

## Methods

### Trial Data Sets

A pooled analysis of 11,052 visits from both arms of 3 randomized, double-blind, placebo-controlled trials in PMS, all with a neutral outcome: Safety and efficacy of MD1003 in progressive multiple sclerosis (SPI-2),^32^ Multiple Sclerosis-Secondary Progressive Multi-Arm Randomisation Trial (MS-SMART),^33^ and Multiple Sclerosis - Simvastatin Trial 2 (MS-STAT2).^34,35^ Individual trial details are summarized in the supplementary material (eTable 1), and full details are reported in their respective publications. For standardized analysis across all clinical trials, we included visits at 6-month visits from baseline, up to a maximum follow-up of 36 months. Data from visits that occurred outside of these intervals were excluded, with the exception of MS-QoL-54 data, for which the only available earlier timepoint was 15 months from baseline.

### Outcome Measures and Definitions

#### EDSS

EDSS confirmed disability progression (CDP) was defined as an increase of at least 1 point for a baseline EDSS ≤5.5 or an increase of at least 0.5 points for a baseline ≥6.0, that was sustained at the next available visit ≥6 months later, calculated until 36 months.

#### PROMs

The MS-QoL-54 is a disease-specific 54-item PROM adapted from the Short Form 36 to include questions relevant to people with MS, resulting in 12 subscores, and 2 composite scores, all scaled out of 100. Of these, the PHC summary score incorporates 8 subscores (physical function, physical role limitation, pain, energy/fatigue, health perceptions, social and sexual function, and health distress); their composite selection and relative weighting are determined by factor analysis, and higher scores indicate a *better* QoL.^25^ The MSIS-29v2 is a 29-item 4-response Likert scale MS-specific PROM, yielding 2 subscores: physical (responses from 20 items, range 20–80) and mental health (responses from 9 items, range 9–36); these are scaled out of 100, with higher scores indicating *worse* function.^26,27^ The MSWS-12v2 is a 12-item MS-specific and mobility-specific PROM with Likert scale responses ranging from 1 to 5, resulting in a single score (range 12–54) scaled out of 100, with a higher score indicating a *worse* level of function.^28,29^ In the cases of the MS-QoL-54 and MSIS-29v2 the PHC and PH subscores, respectively, were used given evidence that physical subscores most closely associate with clinical measures of disability.^23,36,37^ Therefore, the other potential individual subscores (various for the MS-QoL-54 and psychological for the MSIS-29v2) were not included in the analysis. For ease of reporting and interpretation of our analysis, change in the MS-QoL-54 PHC was inverted so that an increase in PHC reflects a worsening QoL; in this way, an *increase* in the scores for any reported PROM reflects *worsening* function.

For PROMs changes, the earliest available follow-up time point in which the PROM was collected was used; this was 15 months for the MS-QoL-54 PHC and 12 months for the MSIS-29v2 PH and MSWS-12v2. Given that the most crucial element of the interim outcome is its capacity to detect a treatment effect, PROM change was analyzed as a continuous measure, retaining full data granularity and variability, and maximizing statistical power and sensitivity to the main outcome association and to the detection of treatment effects.^38^

To assess whether a combination of the MSIS-29v2 PH and MSWS-12v2 could be more robust to floor/ceiling effects and provide stronger association with EDSS, we generated *Z*-scores for either PROM before combining them. This could not be performed for the MS-QoL-54 because this was not collected at the same time point, nor in all the used trial data sets. A *Z*-score is a standardized score representing the number of SD units a given value is from a population mean, it is calculated by subtracting the mean of the whole cohort from the test score then dividing by the SD of the entire cohort. This process allows the different measures to be combined in a similar fashion to the previously established MS Functional Composite.^39^ In the case of MSIS-29v2 PH and MSWS-12v2, an increasing *Z*-score indicated worsening disability, as did a normal score. After generating *Z*-scores for each available PROM, the final composite score consisted of the *Z*-score from the PROM that demonstrated the greatest absolute change for each individual; when only 1 PROM was available, this was used. This approach acknowledges that different PROMs vary in sensitivity to change and places value on the score that demonstrated the most prominent deterioration, avoiding information loss from ceiling effects occurring from a single measure or their direct combination as an average. This mirrors the “and-or” logic used in the PMS trial relevant EDSS-Plus composite, which maximizes sensitivity to disease progression by defining significant worsening as change beyond the thresholds of any of the EDSS, timed 25-foot walk, or 9-hole peg test, rather than conditioning progression on their combined change.^40^

### Statistical Analysis

Summary statistics for EDSS, PROM scores, and follow-up durations are reported as median and interquartile range (IQR), *Z*-scores, and all others as mean and SD unless otherwise specified. Estimated differences in change in PROMs between those that did and did not experience CDP were calculated using the Wilcoxon rank-sum test. The prediction of CDP by PROMs was assessed using Cox proportional hazards, with PROM change as a linear term in the hazard function. We conducted both unadjusted and adjusted model, with the latter including baseline EDSS (≤5.0 vs ≥5.5), baseline PROM (≤39 vs 40 to 69 vs ≥70), age, sex (female vs male), and duration of PMS, and stratified by trial because of different follow-up schedules. For ease of reporting and comparison between PROMs and the composite, the association with EDSS is reported as the relationship between a 1 SD change in PROM and EDSS risk. Missing data were not imputed, and no subgroup or sensitivity analyses were undertaken.

### MAMS Trial Power Analysis: Simulations

For all PROMs with a positive association with CDP, we assessed what MAMS-relevant sample size would be needed to detect realistic treatment effects with sufficient power by simulating MAMS trials. We simulated 10,000 such trials and evaluated the type I and type II error rates at stage 1 interim analyses for hypothetical treatment arms that would prove effective or ineffective at stage 2. For each PROM, we simulated treatment effects (ranging from a 40% to 60% reduction in mean PROM score at the interim time point^30^), incorporated correlation structures between PROM and time to EDSS CDP extracted from our data set, and with varying sample sizes appropriate for a MAMS trial. As a benchmark, we performed the same simulation for annual MRI percentage WBA using expected change parameters from the original multi-trial systematic review used in the design of the OCTOPUS trial,^11^ with a control rate of 0.55%, and estimated SD at 1 year of 0.8%. Although the expected mean percentage reduction in WBA by an effective treatment is 27%, for ease of comparability with PROMs, we simulated the same percentage mean reduction treatment effects on WBA as for PROMs and reported this lower treatment effect in the supplementary material (eTable 2). Each trial simulation consisted of 1 placebo arm and 2 treatment arms (1 effective and 1 ineffective, at stage 2). Interim treatment effects were tested using linear regression with a 1-sided α of 0.35, targeting a more stringent power of 0.95—as per the original OCTOPUS design.^11^

R (version 4.4.1) was used for all computations. Descriptive 2-sided *p*-values are reported and referred to as statistically significant if smaller than 0.05. The methodological steps are presented in Figure 1.

**Figure 1:**
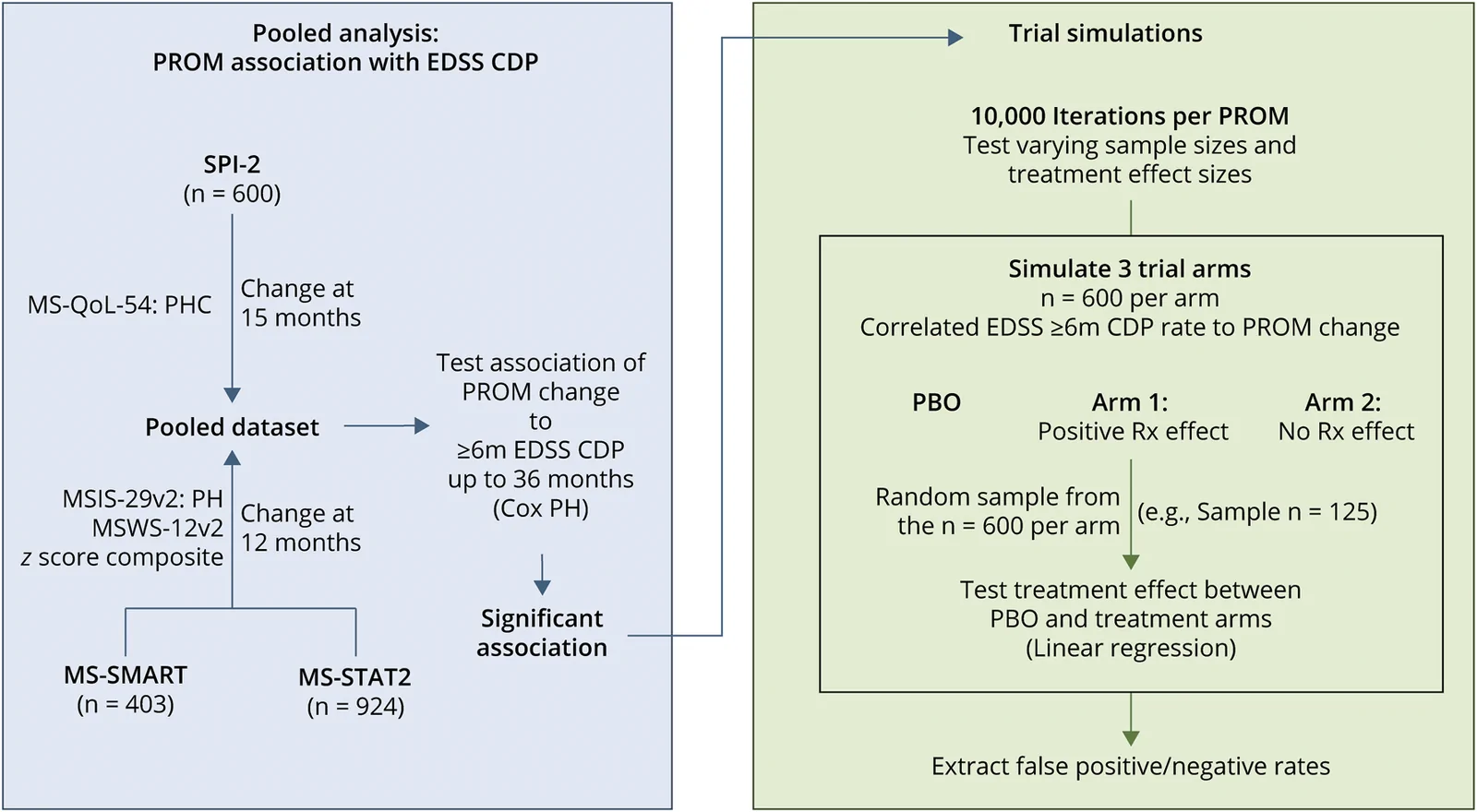
Methods Pooled analysis: Visits across the SPI-2 trial, MS-SMART, and MS-STAT2 were pooled, with 15-month change in the MS-QoL-54: PHC from SPI-2 and 12-month change in the MSIS-29v2 PH, MSWS-12v2, and their composite from MS-SMART and MS-STAT2. Their association with ≥6m EDSS CDP up to 36 months was assessed through Cox proportional hazard testing. Power calculations using trial simulations were performed in any PROM with a significant association with EDSS ≥6m CDP. Trial simulations: For every PROM in this analysis, 3 trial arms with n = 600 sample size were simulated: a placebo arm, an effective treatment arm, and an ineffective treatment arm. In each arm, correlated time to EDSS 6m CDP and interim PROM change were simulated, and a random interim sample of different sizes drawn from the total number. Linear regression between the PBO arm and either other arm was conducted to test for a treatment effect on interim PROM change. This was performed 10,000 times per PROM, interim sample size, and treatment effect size, and the significance/power values extracted. ≥6m EDSS CDP = ≥6-month Expanded Disability Status Scale Confirmed disability progression; CDP = confirmed disability progression; Cox PH = cox proportional hazard testing; EDSS = Expanded Disability Status Scale; MSIS-29v2: PH = Multiple Sclerosis Impact Scale 29 version 2: Physical Health subscore; MS-QoL-54: PHC = Multiple Sclerosis Quality of Life 54: Physical Health Composite; MSWS-12v2 PH = Multiple Sclerosis Walking Scale 12 version 2; PBO = placebo; PROM = patient-reported outcome measure; Rx effect = treatment effect.

### Standard Protocol Approvals, Registrations, and Patient Approvals

The study was conducted in accordance with the Declaration of Helsinki and International Council for Harmonization Good Clinical Practice. For SPI2, data were gathered under the auspices of the International Progressive MS Alliance, and the Institutional Review Board at the Montreal Neurological Institute, Quebec, Canada, approved this study (Reference No. IRB00010120). Ethical approval details in the SPI2 (NCT02936037), MS-SMART (NCT01910259), and MS-STAT2 (NCT03387670) trials are described in the original publications.^32,33,41^ Participants had given written informed consents at the time of trial enrolment.

### Data Availability

The data from MS-SMART and MS-STAT2 used in this study are available on appropriate requests from the chief investigator Prof. J. Chataway (j.chataway@ucl.ac.uk), after 12 months of the initial publication. Data from SPI2 are available on request from the International Progressive MS Alliance data repository.

## Results

### Demographics and Outcome Change

Of the 2,046 participants available, 119 had only baseline data available and were excluded from the analysis; the summarized details are presented in Table 1, and details of the individual trials are presented in the supplementary material (eTable 1). Of the final 1,927 participants, n = 403 were from MS-SMART, n = 600 from SPI2, and n = 924 from MS-STAT2. n = 1,272 (66.0%) were female, with a mean age of 54.0 (SD 7.2) years, duration of progression of 7.5 years (SD 5.6), n = 214 (11.1%) had a diagnosis of primary PMS, and n = 1,713 (88.9%) had SPMS, with a median follow-up duration of 24 months (IQR 18 months), up to 36 months. The median baseline MS-QoL-54 PHC was 47.5 (IQR 21.0), MSIS-29v2 PH was 57.5 (IQR 25.0), and MSWS-12v2 was 68.8 (IQR 29.2).

**Table 1:** Baseline Demographics

		Range
Self-reported sex, n (%)		
Female	1,272 (66.0)	—
Male	655 (34.0)	—
Self-reported ethnicity, n (%)		
White or White British	1,835 (95.2)	—
Asian or Asian British	31 (1.6)	—
Black or Black British	29 (1.5)	—
Mixed	12 (0.6)	—
Other/unknown	20 (1.0)	—
Age (y)	54.0 (7.2)	26.0–66.0
Duration of		
MS progression (y)	7.5 (5.6)	0.8–38.9
Follow-up (mo)	24.0 (18.0)	6.0–36.0
MS type, n (%)		
SPMS	1,713 (88.9)	
PPMS	214 (11.1)	
EDSS	6.0 (1.0)	3.5–6.5
Participants with EDSS ≤5.0	468 (24.3)	
Participants with EDSS ≥5.5	1,459 (75.7)	
PROMs (points out of 100)		
MS-QoL-54: PHC	47.5 (21.0)	0.0–88.7
MSIS-29v2: PH	57.5 (26.3)	6.7–98.3
MSWS-12v2	68.8 (29.2)	4.2–100.0

Abbreviations: EDSS = Expanded Disability Status Scale; IQR = interquartile range; MS = multiple sclerosis; MSIS-29v2: PH = Multiple Sclerosis Impact Scale 29 version 2: Physical Health subscore; MS-QoL-54: PHC = Multiple Sclerosis Quality of Life 54: Physical Health Composite; MSWS-12v2 = Multiple Sclerosis Walking Scale 12 version 2; PPMS = primary progressive multiple sclerosis; PROMs = patient-reported outcomes; SPMS = secondary progressive multiple sclerosis.

Data are n (%) for categories, mean (SD) for continuous measures, and median (IQR) for EDSS, follow-up duration, and PROMs.

CDP occurred in 495 participants (25.7%), corresponding to an annualized event rate of 0.582 events per patient-year (32.4% cumulative event risk over 36 months). In the whole cohort, the median MS-QoL-54 PHC at 15 months increased by 0.39 points (IQR 13.66), MSIS-29v2 PH at 12 months by 1.25 points (IQR 18.33), and MSWS-12v2 at 12 months by 0 points (IQR 14.58). The estimated mean differences in PROM change between those that experienced CDP and those that did not were 1.92 points (−0.68 to 4.52, *p* = 0.15) in the MS-QoL-54 PHC at 15 months, 1.67 points (0.00–3.33, *p* = 0.07) in the MSIS-29v2 PH at 12 months, and 0.15 points (0.06–0.25, *p* < 0.01) in the MSWS-12v2 at 12 months.

### Association of PROMs With EDSS ≥6-Month CDP

Change in the MS-QoL-54 PHC at 15 months was not associated with risk of CDP (hazard ratio [HR] 1.14 [0.91–1.42], *p* = 0.24).

A 1 SD increase conveyed an increased CDP risk in the MSIS-29v2 PH (HR 1.20 [1.08–1.33], *p* < 0.0001), MSWS-12v2 (HR 1.26 [1.13–1.40], *p* < 0.0001), and composite (HR 1.29 [1.16–1.44], *p* < 0.0001). Statistical significance was retained in the adjusted models for all these PROMs (MSIS-29v2 PH: 1.34 [1.20–1.49], *p* < 0.0001; MSWS-12v2: 1.43 [1.26–1.63], *p* < 0.0001; composite: 1.48 [1.31–1.68], *p* < 0.0001). The predicted increase in hazard conveyed by PROM per-point change is presented in Figure 2A and exemplified in estimated survival curves divided into percentiles in Figure 2B.

**Figure 2:**
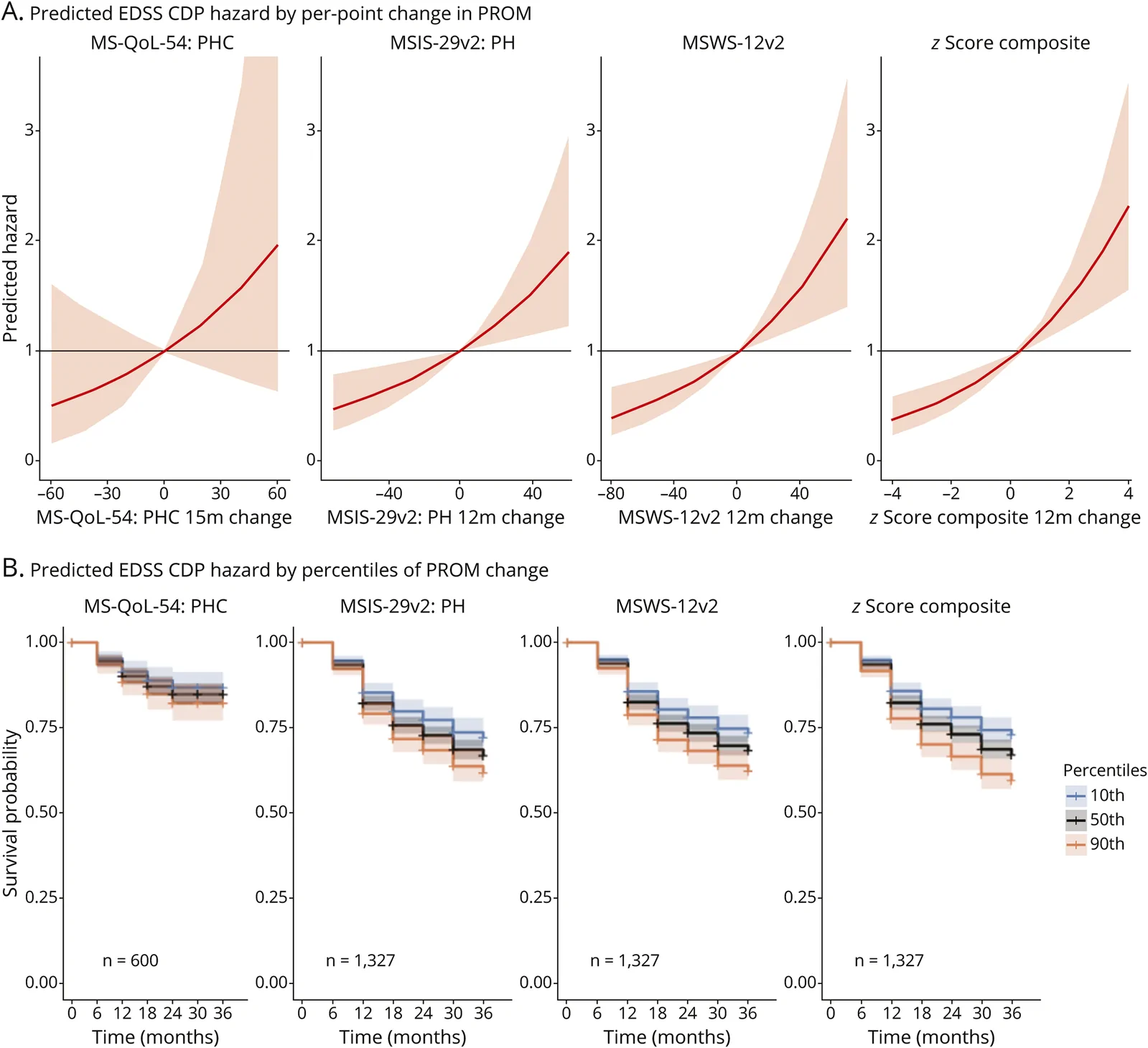
Model-Predicted Impact of PROM Changes on EDSS CDP HRs (A) HR plot showing the predicted risk of EDSS CDP per 1-point change in the PROMs (solid red line, 95% CI in light red); the solid black line indicates no association (HR = 1). TheMSIS-29v2 PH, MSWS-12v2, and their composite showed a significant association with EDSS CDP, while the MS-QoL-54 PHC did not. (B) Kaplan-Meier Curves illustrating the predicted EDSS CDP risk across PROM change percentiles (10th, 50th, and 90th), with the corresponding patient numbers in each analysis indicated. The 10th percentile (blue line) represents the lowest reported worsening, the 50th percentile (black line) the median, and the 90th percentile (red line) represents the greatest reported worsening. CDP = confirmed disability progression; EDSS = Expanded Disability Status Scale; HR = hazard ratio; MSIS-29v2: PH = Multiple Sclerosis Impact Scale 29 version 2: Physical Health subscore; MS-QoL-54: PHC = Multiple Sclerosis Quality of Life 54: Physical Health Composite; MSWS-12v2 = MS Walking Scale 12 version; PROM = patient-reported outcome.

### MAMS Trial Power Analysis: Simulations

Because the MS-QoL-54 PHC did not show a significant association with CDP, trial simulations were not conducted for this PROM. Comparing results across sample sizes and treatment effects (Table 2), with a sample size of 125/arm and simulated 40% treatment effect, the percentage power for the MSIS-29v2 PH was 68.8% (59.2–77.0), with a sample size of 200/arm; a simulated 60% treatment effect resulted in a limited improvement to 69.1% (59.5–77.3). The MSWS-12v2 showed marginally greater statistical power of 70.7% (61.1–78.7) and 73.7% (64.4–81.4), respectively.

**Table 2:** Trial Simulation Results

Mean PROM change treatment effect	Interim outcome sample size per arm (n)	Type I error rate alpha (%)	Power (%)
40% reduction			
Annual percent MRI whole-brain atrophy	125	17.6 (11.4–26.3)	98.2 (93.3–99.5)
	150	17.1 (11.0–25.6)	98.7 (94.1–99.7)
	175	17.5 (11.3–26.1)	99.1 (94.7–99.9)
	200	17.4 (11.2–26.0)	99.4 (95.3–99.9)
MSIS-29v2 PH	125	17.6 (11.4–26.2)	68.8 (59.2–77.0)
	150	18.0 (11.7–26.6)	68.9 (59.3–77.2)
	175	17.7 (11.5–26.3)	68.8 (59.2–77.0)
	200	17.9 (11.7–26.6)	69.2 (59.6–77.4)
MSWS-12v2	125	17.1 (10.9–25.6)	70.7 (61.1–78.7)
	150	17.6 (11.4–26.2)	71.0 (61.5–79.0)
	175	17.5 (11.3–26.1)	71.4 (61.8–79.3)
	200	17.8 (11.6–26.5)	71.5 (62.0–79.5)
Composite	125	17.3 (11.1–25.9)	87.2 (79.2–92.4)
	150	17.5 (11.3–26.1)	88.4 (80.6–93.3)
	175	17.6 (11.3–26.2)	90.3 (82.9–94.7)
	200	18.1 (11.8–26.8)	91.4 (84.3–95.5)
50% reduction			
Annual percent MRI whole-brain atrophy	125	17.7 (11.4–26.3)	99.4 (95.3–99.9)
	150	17.4 (11.2–26.0)	99.8 (95.8–100)
	175	17.3 (11.1–25.9)	99.9 (96.1–100)
	200	17.7 (11.5–26.3)	99.9 (96.2–100)
MSIS-29v2 PH	125	17.8 (11.5–26.4)	68.7 (59.1–77.0)
	150	17.3 (11.1–25.8)	69.1 (59.5–77.3)
	175	17.5 (11.3–26.2)	68.9 (59.2–77.1)
	200	17.5 (11.3–26.1)	68.9 (59.3–77.2)
MSWS-12v2	125	17.7 (11.5–26.4)	71.6 (62.1–79.5)
	150	17.9 (11.6–26.5)	71.7 (62.2–79.6)
	175	17.5 (11.3–26.1)	72.2 (62.7–80.0)
	200	17.5 (11.3–26.1)	72.8 (63.3–80.5)
Composite	125	17.5 (11.3–26.1)	90.9 (83.7–95.1)
	150	17.6 (11.4–26.3)	92.6 (85.7–96.3)
	175	17.6 (11.4–26.3)	93.8 (87.3–97.1)
	200	17.8 (11.5–26.4)	95.0 (88.8–97.8)
60% reduction			
Annual percent MRI whole-brain atrophy	125	17.2 (11.1–25.8)	99.9 (96.1–100)
	150	16.8 (10.7–25.3)	100 (96.3–100)
	175	17.4 (11.3–26.1)	100 (96.3–100)
	200	17.2 (11–25.7)	100 (96.3–100)
MSIS-29v2 PH	125	17.6 (11.4–26.3)	69.1 (59.5–77.3)
	150	17.2 (11.1–25.8)	70.0 (60.4–78.1)
	175	17.6 (11.4–26.2)	69.4 (59.8–77.6)
	200	17.2 (11.1–25.8)	69.1 (59.5–77.3)
MSWS-12v2	125	17.8 (11.5–26.4)	72.3 (62.9–80.2)
	150	17.3 (11.1–25.9)	72.2 (62.7–80.0)
	175	17.4 (11.2–26.0)	73.2 (63.8–80.9)
	200	17.3 (11.1–25.9)	73.7 (64.4–81.4)
Composite	125	17.3 (11.2–25.9)	94.2 (87.7–97.3)
	150	17.7 (11.5–26.3)	95.2 (89.2–98.0)
	175	17.6 (11.4–26.2)	96.2 (90.4–98.5)
	200	17.5 (11.3–26.2)	97.2 (91.9–99.1)

Abbreviations: MSIS-29v2 PH = Multiple Sclerosis Impact Scale 29 version 2 physical health subscore; MSWSv2 = Multiple Sclerosis Walking Scale 12 version 2; PROM = patient-reported outcome measure.

This table lists simulated treatment effects on PROMS of interest, with annual MRI whole-brain atrophy as a benchmark, and resultant percentage false-negative and false-positive rates over different sample sizes. Assuming a 50% mean reduction treatment effect, the composite achieves sufficient power with a sample size of 200 participants.

The *Z*-score composite measure demonstrated improved error rates and responsiveness to sample sizes and treatment effects, with a corresponding percentage power of 87.2% (79.2–92.4) and 97.2% (91.9–99.1), respectively.

## Discussion

In this study, using a large, well-characterized data set of patients with PMS (>2,000), we have demonstrated that change in the MS-QoL-54 PHC score was not associated with CDP risk, whereas 12-month changes in the MSIS-29v2 PH, MSWS-12v2, and their composite *Z*-scores were associated with 36-month EDSS CDP risk. Using trial simulations, we have calculated that when used individually, the MSIS-29v2 PH and MSWS-12v2 did not have sufficient power to act as sole interim outcome measures, with limited gains despite increasing sample sizes and treatment effects. However, the 12-month change in the composite *Z*-score of the MSIS-29v2 PH and MSWS-12v2 demonstrated high power for treatment effect detection: a 50% reduction being seen at powers of 90% (125/arm) and 97% (200/arm).

Furthermore, future studies could assess the potential gains of incorporating both MRI and PROMs into a multimodal interim outcome to take advantage of their respective strengths, with MRI providing a biological measure and PROMs providing a patient-derived, cost-effective measure of function that can be collected remotely. This combination may improve conditional power and information accrual, enabling further reductions in sample sizes or earlier interim decision making.^42-44^

There have been increasing efforts by regulators and research communities to meaningfully include PROMs in clinical trials.^45-48^ However, the application of these efforts has been hindered by 2 major overlapping challenges: uncertainty regarding which PROMs are adequately responsive to quality of life in pwMS, and second, how to interpret their longitudinal change meaningfully for treatment effects. Over 400 PROMs have been used in MS research, of which 82 have been developed specifically for pwMS,^49^ with a broad uncertainty about which are psychometrically and conceptually suitable for the outcome target in question.^49-51^ Compounding this, although there has been greater consistency in PROM selection for PMS trials in the form of the MSIS-29, MSWS-12, and MS-QoL-54,^4^ reporting is typically limited to mean change from baseline to end of study. Using this approach, the few trials that have demonstrated a positive clinically meaningful benefit in PMS have failed to do the same for their respective PROMs.^31,52,53^

This apparent inconsistency between clinical and patient-reported outcomes however is likely belied by a complex combination of factors, including the impact of baseline values conveying floor and ceiling effects on change (with the latter being particularly salient in PMS trials),^54,55^ that the change itself captures variations in *perceived* quality of life which can also improve over time as an individual becomes more familiar with their initial or new physical disabilities (“response shift”) in a fashion that is disparate from clinical measures of impairment that invariably worsen over time,^56-59^ and the influence of important demographic, socioeconomic, and disease-specific factors.^24,60^

In contrast to the challenges described, there is clear evidence, as we present here, that PROM value should not be dismissed. Studies have shown clear relationships in early PROM scoring in PMS compared with relapse-onset MS,^61,62^ the transition from relapsing to PMS,^51,62,63^ between PROM scores to disability progression,^23,64^ and evidence that worse PROM scores predict risk of mortality independently of physical measures of disability.^65^ Our positive findings with the MSIS-29v2 PH and MSWS-12v2, and their composite, build on evidence of their ability in trial to detect treatment effects when restricting follow-up to shorter durations,^66,67^ which is likely a reflection of their ability to demonstrate a treatment effect without losing signal from mean change to perceptual “response shift” over longer periods.^56-58^ This is appreciable in a post hoc phase 3 trial analysis of the MSIS-29v2 and MSWS-12v2 by Hobart et al., which demonstrated a clear treatment effect in mean PROM change at 12 months, with a significant reduction in mean change in the MSIS-29v2 PH of 70% and in the MSWS-12v2 of 55%, which was clearly attenuated at 24 months.^30,54^

Therefore, we sought a way to overcome the challenges of ceiling effects and response shift through a composite. Toward this end, we drew from the designs of established PMS clinical outcomes, specifically the MS Functional Composite^68^ and EDSS-Plus,^40^ which combine complementary measures of disability. Akin to the MS Functional Composite, we used *Z*-scoring to combine the broader physical disability data from the MSIS-29v2 PH and the mobility-specific MSWS-12v2 into a unified scale. However, a direct average of the 2 scores would risk the ceiling effect of 1 PROM diluting a significant change in another. Therefore, drawing from the EDSS-Plus which defines disability progression as significant worsening in *any one* of its composite elements, our *Z*-score composite selects the PROM value with the greatest degree of change, allowing it to convey the clearest value of increasing disability from either available PROM.

Our study has several limitations. Our focus on PMS trials precludes the generalizability of our findings to relapsing remitting MS,^69^ and the trial populations in MS-SMART and MS-STAT2 enrolled people with SPMS. The EDSS and PROM collection intervals, primary outcome, and maximum follow-up durations differed between studies, which may influence effect estimates.

In conclusion, our findings build on earlier work in identifying early markers related to established trial and regulator relevant final outcome measures and support the use of PROMs as interim outcome measures, particularly as composite measures.

## Glossary

CDP: confirmed disability progression
EDSS: Expanded Disability Status Scale
HR: hazard ratio
IQR: interquartile range
MAMS: multi-arm multistage
MSIS-29v2 PH: MS Impact Scale 29 version 2 physical health subscore
MS-QoL-54 PHC: MS Quality of Life 54 Physical Health Composite
MSWS-12v2: MS Walking Scale 12 version 2
PMS: progressive multiple sclerosis
PROM: patient-reported outcome
QoL: quality of life
SPMS: secondary progressive MS
WBA: whole-brain atrophy
